# Maternity and Child Welfare

**Published:** 1920-01-24

**Authors:** 


THE PUBLIC WELL-BEING—(*continued*)

### MATERNITY AND CHILD WELFARE.

The Maternity and Child Welfare Act, 1918, removed any difficulty which local authorities might have felt as to their legal powers to incur expenditure for purposes of maternity and child welfare. Some action has been undertaken by all county councils and all metropolitan boroughs, and the report of the Local Government Board just issued says there are very few of the smaller authorities which have not come into a county scheme or are not carrying on work of their own. The extension of the work undertaken by the county councils and larger urban authorities has led to the engagement of a number of whole-time assistant medical officers of health for this work only, or for this work in combination with that connected with school, housing schemes, preventive medicine, tuberculosis, venereal disease, and other medical undertakings.

